# Sociogenomics of Cooperation and Conflict during Colony Founding in the Fire Ant *Solenopsis invicta*

**DOI:** 10.1371/journal.pgen.1003633

**Published:** 2013-08-08

**Authors:** Fabio Manfredini, Oksana Riba-Grognuz, Yannick Wurm, Laurent Keller, DeWayne Shoemaker, Christina M. Grozinger

**Affiliations:** 1 Department of Entomology, Center for Pollinator Research and the Huck Institutes of the Life Sciences, Pennsylvania State University, University Park, Pennsylvania, United States of America; 2 Department of Ecology & Evolution, University of Lausanne, Lausanne, Switzerland; 3 USDA-ARS, Gainesville, Florida, United States of America; University of California-Berkeley, United States of America

## Abstract

One of the fundamental questions in biology is how cooperative and altruistic behaviors evolved. The majority of studies seeking to identify the genes regulating these behaviors have been performed in systems where behavioral and physiological differences are relatively fixed, such as in the honey bee. During colony founding in the monogyne (one queen per colony) social form of the fire ant *Solenopsis invicta*, newly-mated queens may start new colonies either individually (haplometrosis) or in groups (pleometrosis). However, only one queen (the “winner”) in pleometrotic associations survives and takes the lead of the young colony while the others (the “losers”) are executed. Thus, colony founding in fire ants provides an excellent system in which to examine the genes underpinning cooperative behavior and how the social environment shapes the expression of these genes. We developed a new whole genome microarray platform for *S. invicta* to characterize the gene expression patterns associated with colony founding behavior. First, we compared haplometrotic queens, pleometrotic winners and pleometrotic losers. Second, we manipulated pleometrotic couples in order to switch or maintain the social ranks of the two cofoundresses. Haplometrotic and pleometrotic queens differed in the expression of genes involved in stress response, aging, immunity, reproduction and lipid biosynthesis. Smaller sets of genes were differentially expressed between winners and losers. In the second experiment, switching social rank had a much greater impact on gene expression patterns than the initial/final rank. Expression differences for several candidate genes involved in key biological processes were confirmed using qRT-PCR. Our findings indicate that, in *S. invicta*, social environment plays a major role in the determination of the patterns of gene expression, while the queen's physiological state is secondary. These results highlight the powerful influence of social environment on regulation of the genomic state, physiology and ultimately, social behavior of animals.

## Author Summary

The characterization of the genomic basis for complex behaviors is one of the major goals of biological research. The genomic state of an individual results from the interplay between its internal condition (the “nature”) and the external environment (the “nurture”), which may include the social environment. Colony founding in the fire ant *Solenopsis invicta* is a complex process that serves as a useful model for investigating how the interplay between genes and social environment shapes social behavior. Unrelated, newly mated *S. invicta* queens may start a new colony as a group, but ultimately only one queen will survive and gain full reproductive dominance. By uncovering the genetic basis for founding behavior in fire ants we therefore provide useful insights into how cooperative behavior evolved in a context that might be considered primitively eusocial, because newly mated queens in a founding association are morphologically, physiologically and genetically very similar and display no evident division of labor. Our results suggest that social environment (founding singly or in pairs, switching dominance rank vs. maintaining rank) is a much greater driver of gene expression changes than social rank itself, suggesting that social environment, and not reproductive state, is a key regulator of gene expression, physiology and ultimately, behavior.

## Introduction

Behavior is a complex phenotypic trait, which results from the interactions of multiple intrinsic and extrinsic factors that associate in a nonlinear, often unpredictable fashion [1]. Intrinsic factors include the genetics, the physiology or the phenotype of an organism, while the most typical extrinsic factor is the external environment. In social systems like insect societies, environmental cues primarily are the result of the social environment, i.e. the nature and patterns of interactions among individuals within the colony [2]. The “nature-versus-nurture” debate has long been the major driver of the discussion as to whether internal state of an animal or the external environment (e.g., the social environment) regulates gene expression more [3]. Regardless, extrinsic and intrinsic factors clearly are reciprocally interconnected: the social environment influences the neurogenomic state of the animal, which is responsible for the social behavior performed [4], [5]. A hallmark of advanced social behavior is altruistic behavior, which is achieved through a reproductive division of labor in which few individuals develop into the reproductive caste while most of the colony members become non-reproductive workers and perform all tasks related to colony maintenance and growth. Both fixed (developmental pathways) and plastic (behavioral strategies) factors contribute to this division of labor (reviewed in [6]). Consequently, there has been great interest in studying genes and biological processes that regulate the reproductive and worker divisions of labor [7]. In the advanced eusocial systems examined thus far, differences between queens and workers are largely the result of developmental factors, while differences among workers are often triggered by social signals [8]. However, primitively social systems display reproductive division of labor between females that are anatomically, physiologically and genetically very similar and this reproductive division of labor seems to be primarily established and maintained by social environment. The genes underlying this process have not yet been examined, and potentially may function as core genes associated with sociality.

Variation in colony founding among ant queens is an ideal model to examine the interplay between genes and social environment that has shaped the evolution of cooperative behavior in primitively social systems. Colony founding can occur in two modalities: haplometrosis, where a single queen independently starts a new colony, and pleometrosis, where multiple queens associate and cooperate to start a new colony [9]. Pleometrosis is a fascinating example of cooperative behavior that is not fostered by kin selection, because these groups often comprise unrelated individuals (reviewed [10]). Among social insects, pleometrosis exists in halictine bees [11], termites [12], paper wasps [13], [14], [15] and in several species of ants [8]. In ants, pleometrosis is known to be associated with division of labor in the leaf-cutter ant *Acromyrmex versicolor* [16] and in the harvester ant *Pogonomyrmex californicus* [17]. Pleometrotic associations produce a complex social environment, where individuals simultaneously are in cooperation and conflict, and social and reproductive dominance hierarchies are established. These associations represent relatively primitive social systems in which individuals with equivalent anatomical and physiological features develop a division of labor through their behavioral interactions. Thus, identification of the genes underlying establishment of these hierarchies will not only provide insight into the effects of social environment on an individual, but also into the evolution of social behavior.

The red imported fire ant *Solenopsis invicta* is an excellent system for studying the genes associated with haplometrotic and pleometrotic behaviors, because queens from the monogyne social form (characterized by a single egg-laying queen per nest once established) can adopt either approach for colony founding depending on multiple factors, e.g. the density of newly mated queens in nesting sites [18]. However, monogyne fire ants ultimately only tolerate a single reproductive queen such that the initial cooperation among unrelated pleometrotic cofoundresses slowly transitions to competition and rivalry, which will inevitably produce only one winner and one or multiple losers [19], [20], [21]. Once the first workers emerge, pleometrotic queens engage in open fights where they injure or kill rival cofoundresses and workers actively participate in this process until all the queens are executed but one (see Movie S1). In both haplometrosis and pleometrosis, founding queens initially face a critical period (claustral period) where they are sealed in their nest and must defend it from enemies and competitors, e.g. other fire ant colonies that populate the same area [22], [23]. During the claustral period, fire ant queens rely exclusively on their body mass reserves to produce the first generation of workers. There are physiological and behavioral differences between haplometrotic queens, pleometrotic “winners” and pleometrotic “losers”. Haplometrotic queens lose more weight during the claustral period, and produce more brood per individual than queens in pleometrotic associations [24], [25]. In pleometrotic associations, winners tend to have larger head size, lose less weight [20], [21], [26] and occupy the top of the brood pile while losers are usually found outside the nest chamber [19], attempting to avoid any interaction with the winner or with workers. However, nothing is known about the genes and molecular pathways that underlie these processes.

We performed two separate experiments to characterize the genomic basis for haplometrotic and pleometrotic founding behavior in fire ants. We developed a microarray platform using the official gene set of the fire ant genome [27] plus a set of ESTs obtained from assemblies of the fire ant transcriptome to examine genome-wide expression patterns across founding queens. In the first experiment, we compared whole body gene expression patterns among haplometrotic queens and paired pleometrotic winners and losers that were collected shortly after emergence of the first workers (but prior to execution of the loser). We predicted that haplometrotic queens would be more similar to pleometrotic winners than to pleometrotic losers, because they both will serve as the single queen for the mature colony. For the comparison between winners and losers alone, our expectations were less well-defined: on one hand, we expected to find substantial differences given that their physiology, behavior and fate differ significantly, but on the other hand, winners and losers are not anatomically distinct and there is only weak correlation between morphological measures and outcome of the conflict [20]. In the second experiment, we manipulated queen rank in pleometrotic pairs to determine how changing social rank and social environment would affect an individual's gene expression patterns. This was accomplished by pairing pleometrotic queens with a new partner at the end of the claustral period in order to switch putative winners to losers and vice-versa. Controls in which partners were altered (and social environment was changed) but social rank remained the same were also included. We hypothesized that final social rank would be the primary regulator of gene expression patterns. However, for both experiments our results indicated that social environment (pleiometrosis vs. haplometrosis, switched rank vs. maintained rank) was a much greater driver of gene expression changes than social rank itself, suggesting that social environment, and not reproductive state, is a key regulator of gene expression, physiology and ultimately, behavior.

## Results

### Experiment 1: Effect of social environment and social rank on global gene expression patterns

**Figure 1:**
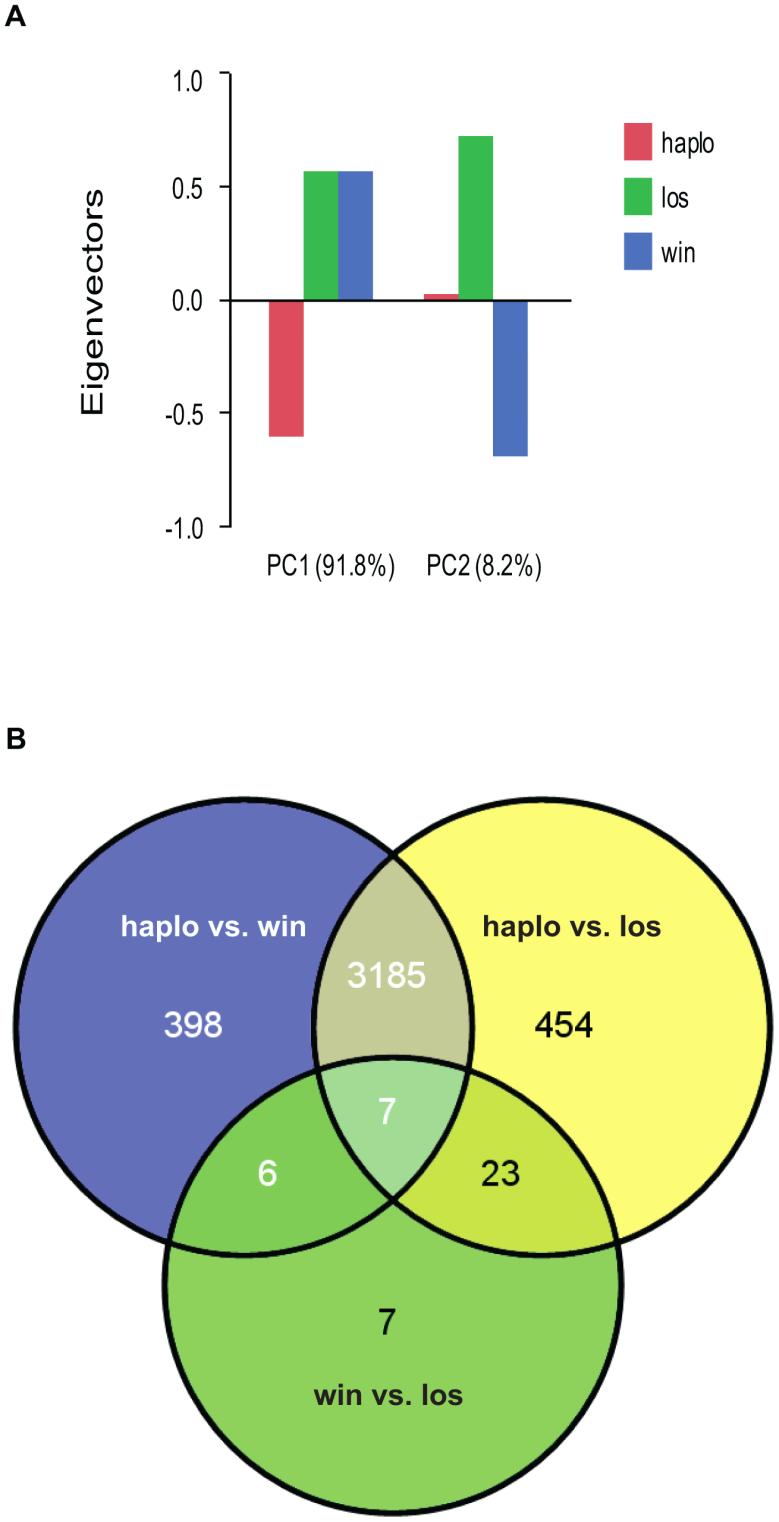
Analyses of global gene expression between haplometrotic and pleometrotic queens. A) Principal Component Analysis of 4080 significantly differentially regulated transcripts. Two PCAs were identified corresponding to the effect of social environment (single queen vs. paired queens, 91.8%) and social rank (winner vs. loser, 8.2%). B) Pairwise comparisons of 4080 significantly differentially regulated transcripts. A total of 3192 transcripts were different between haplometrotic and pleometrotic queens, while only 43 transcripts were different between winner and losers. Haplometrotic queens were more similar to pleometrotic winners than to pleometrotic losers: more transcripts were differentially regulated in haplo vs. los than in haplo vs. win (477 and 404, respectively): this difference was statistically significant (Nominal Logistic Fit: df = 1, ChiSquare = 6.78, P = 0.0092). haplo = haplometrotic queens; los = pleometrotic losers; win = pleometrotic winners.

Haplometrotic queens (haplo) and paired pleometrotic winners (win) and losers (los) were collected shortly after emergence of the first workers (see methods, N = 8 haplo, 8 win and 8 los). Microarray analysis of gene expression patterns (see Methods for design and validation of microarrays) in whole bodies of these queens revealed that 4080 of the 9388 transcripts included in the analysis were differentially regulated at FDR<0.001 (Table S1). A principal components analysis (PCA) of the differentially regulated transcripts revealed that the social environment is more important than social rank in driving the patterns of gene expression in founding queens (Figure 1A). Differences between haplometrotic and pleometrotic queens accounted for 91.8% of the variation in gene expression while differences between win and los accounted for only 8.2%. Pairwise comparisons of transcripts differentially regulated (FDR<0.001) among the three groups of fire ant queens demonstrated that expression patterns in haplo are more similar to win than to los, since there are fewer genes differentially regulated uniquely between haplo and win (404) than haplo and los (477; Nominal Logistic Fit: df = 1, ChiSquare = 6.78, P = 0.0092; Figure 1B).

**Figure 2:**
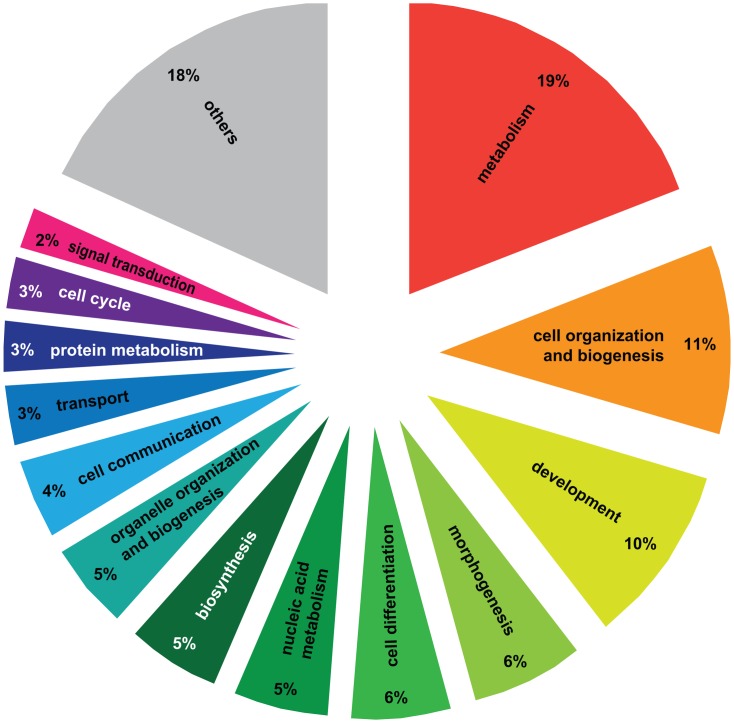
Gene Ontology analysis of genes differentially regulated between haplometrotic and pleometrotic queens. The figure shows the larger functional groups that encompass significantly enriched GO terms resulting from the GO analysis. To obtain this result we overlapped the list of significantly enriched GO terms from experiment 1 (Functional Annotation Chart, P<0.05) with the GO_slim2 list of the cateGOrizer. Others = larger functional groups encompassing less than 2% of the total number of significantly enriched GO terms (see Table S3 for the complete list of ancestor GO terms).

We performed Gene Ontology analysis on the 3003 differentially regulated transcripts (out of the initial pool of 4080) that have *Drosophila* orthologs with FlyBase annotations using DAVID [28], [29]. 517 GO terms were significantly enriched at a p-value<0.05 (Functional Annotation Chart, see Table S2 for the complete list of GO terms). Additionally, 6 KEGG molecular pathways (Kyoto Encyclopedia of Genes and Genomes, [30]) were significantly enriched (P<0.05): aminoacyl-tRNA biosynthesis, basal transcription factors, dorso-ventral axis formation, endocytosis, RNA degradation and ubiquitin mediated proteolysis (Table S2). To cluster the GO categories into larger functional groups, the 517 significantly enriched GO terms were mapped to the GO_slim2 file in CateGOrizer [31]: 440 GO terms were assigned to one of the ancestor terms by single count (Figure 2). The functional groups containing the greatest number of GO terms were metabolism (19% of the significantly enriched GO terms), cell organization and biogenesis (11%) and development (10%; for a complete list of all ancestor terms represented in this analysis see Table S3).

To further characterize the genes that were differentially regulated between haplo and pleometrotic queens (pleo), we examined the overlapping set of 3192 transcripts (of which 2541 had *Drosophila* orthologs with FlyBase annotations) that were differentially regulated between both haplo vs. win and haplo vs. los (Figure 1B). For clearer graphical presentation, we used k-means Clustering in GENESIS [32] to separate these transcripts into two large clusters according to expression patterns: 2280 transcripts that were upregulated in haplo and downregulated in pleo (cluster 1, Figure S1) and 912 transcripts downregulated in haplo and upregulated in pleo (cluster 2, Figure S2). We performed GO analysis on both groups, using Functional Annotation Clustering with medium stringency. For cluster 1 (1925 FlyBase matches), we obtained 88 significantly enriched GO terms (see Table S4; P<0.05) and 1 KEGG pathway (basal transcription factors, P = 0.01). Several of the GO terms were related to aging (determination of adult life span, death), immunity (immune system development, JNK cascade, hemopoiesis), reproduction (reproductive developmental process, oocyte development, eggshell formation, morphogenesis of follicular epithelium, regulation of oocyte development), response to stimuli (response to stress, regulation of response to stimulus, negative regulation of response to stimulus, response to ecdysone), lipid biosynthetic process, locomotion and neurological system processes (neurotransmitter secretion, neurogenesis, central nervous system development, regulation of nervous system development). In cluster 2 (616 FlyBase matches), 34 GO terms (Table S5, P<0.05) and 1 KEGG pathway (glycerophospholipid metabolism, P = 0.01) were significantly enriched. Many GO terms were similar to those in cluster 1, and included determination of adult life span, olfactory behavior, lipid metabolic process, detection of light stimulus, as well as several related to morphogenesis or development of organs and apparatuses like sensory organ, muscle, limb, wing disc, gut and respiratory system.

Interestingly, only 43 transcripts were differentially regulated between win and los queens. A GO analysis performed on this small set of transcripts revealed that fatty acid and hormone metabolic processes were significantly enriched GO terms (Functional Annotation Clustering, P<0.001 and P<0.01, respectively, Table S6). Several transcripts in this group have interesting functions (Table 1). Transcripts upregulated in win included: *G protein-coupled receptor kinase 2* (*Gprk2*), which is involved in the Toll signaling pathway during the response against Gram positive bacteria [33]; *endosulfine* (*endos*), which functions in the insulin-signaling pathway during oogenesis [34]; *Pheromone-binding protein-related protein 3* (*Pbprp3*), a member of the odorant binding proteins responsible for chemoreception [35]; and *bubblegum* (*bgm*), which is involved in the metabolism of very long-chain fatty acids and prevents neurodegeneration [36]. Transcripts upregulated in los relative to win included: *I'm not dead yet* (*Indy*), associated with aging [37]; *pale* (*ple*), which plays a role in the response to wounding [38] and in the metabolism of dopamine [39]; *desat1*, a major regulator of cuticular hydrocarbon biosynthesis involved in pheromone emission and detection [40]; and *juvenile hormone acid methyltransferase* (*jhamt*), a key enzyme in the biosynthesis of JH, the major endocrine regulator in insects [41].

**Table 1:** Patterns of expression of the most relevant transcripts that were differentially regulated between winners and losers in experiment 1 (P<0.001).

Sinv_gene	Dmel_orthologs	GO_terms	winner	loser
SINVlc3_024097	*G protein-coupled receptor kinase 2*	vitellogenesis; defense response to Gram-positive bacterium	up	↓
SINVlc3_031722	*endosulfine*	response to nutrient; water homeostasis; oogenesis	up	↓
SINVlc3_008488	*Probable cytochrome P450 4ac1*	oxidation reduction; monooxygenase activity	up	↓
SI2.2.0_13106	*Cytochrome P450-4c3*	electron carrier activity; oxidoreductase activity	up	↓
SI2.2.0_06972	*Pheromone-binding protein-related protein 3*	odorant binding	up	↓
SINVlc3_031713	*bubblegum*	long-chain fatty acid metabolic process	up	↓
SI2.2.0_03032	*I'm not dead yet*	determination of adult lifespan	↓	up
SINVlc3_027280	*pale*	adult locomotory behavior; dopamine metabolic process; response to wounding	↓	up
SI2.2.0_08208	*Loquacious*	central nervous system development	↓	up
SI2.2.0_00216	*desat1*	pheromone/cuticle hydrocarbon/fatty acid biosynthetic processes	↓	up
SI2.2.0_14785	*Probable phosphomevalonate kinase*	cholesterol biosynthetic process	↓	up
SINVlc3_042741	*juvenile hormone acid methyltransferase*	juvenile hormone biosynthetic process; methylation	↓	up

### Experiment 1: Candidate gene pathways

**Figure 3:**
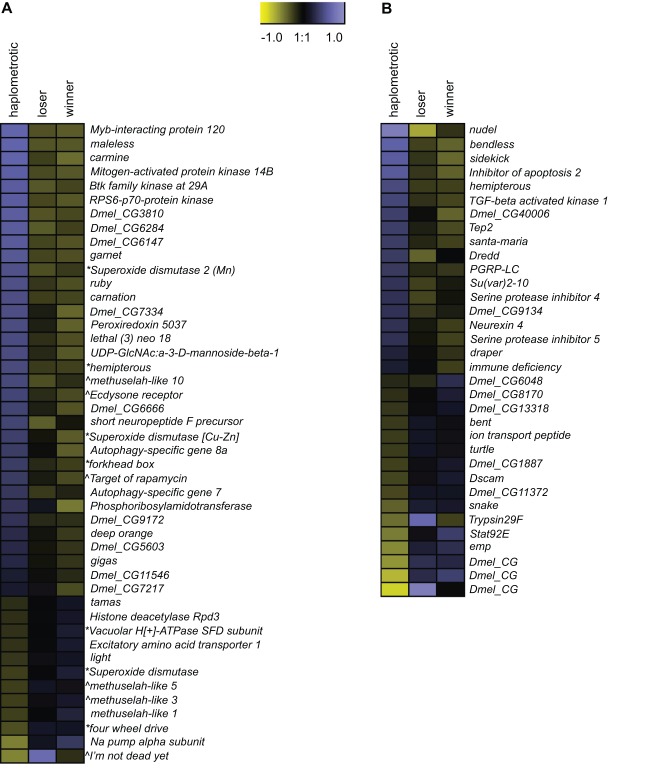
Expression patterns of aging- and immune-associated genes. A) Heatmap of log2 transformed and normalized expression values for the fire ant transcripts that are putatively involved in aging processes. This gene list was obtained by overlapping the list differentially regulated transcripts with FlyBase matches in experiment 1 with the list of genes included in the *Drosophila* GO term “aging”. The directional expression of these transcripts was also compared to the expression patterns of aging genes as found in the review by Paaby and Schmidt [65]. * = gene that extends lifespan when its activity is increased; ∧ = gene that extends lifespan when its activity is decreased. B) Heatmap of log2 transformed and normalized expression values for the fire ant transcripts that are putatively involved in immune processes. This gene list was obtained by overlapping the list differentially regulated transcripts with FlyBase matches in experiment 1 with the list of canonical immune-related genes annotated in honey bees [48].

GO categories related to aging and longevity were significantly enriched in sets of transcripts that were differentially regulated in haplo vs. pleo (clusters 1 and 2; see Tables S4 and S5). Out of the 129 genes included in the *Drosophila* aging GO term, oligos representing 93 putative orthologs were present on the fire ant microarray. Of these, 90 were expressed at high enough levels to be included in the microarray analysis and 46 were significantly differentially regulated across the three groups of queens (Figure 3A). The majority of these genes (34) were upregulated in haplo: this number was significantly higher than expected by chance (Nominal Logistic Fit: df = 2, ChiSquare = 29.58, P<0.0001). In addition to their role in regulating aging pathways in *Drosophila*, several genes in this group have been linked to queen vs. worker caste differentiation and behavioral maturation in honey bees [42]. These include: *forkhead box* (*foxo*) and *target of rapamycin* (*TOR*), two major players in the insulin-signaling pathway which is associated to caste determination in honey bees [43] and to the workers' transition from nursing to foraging behavior [44], [45], and *Peroxiredoxin 5037* (*Prx3*), which is associated with enhanced learning ability when expressed at higher levels in honey bee workers [46].

To further investigate the patterns of expression of aging genes in haplo and pleo queens, we compared our study to a study on aging in *Drosophila* [47]. In this study, which was aimed at investigating the temporal and spatial (tissue-specific) transcriptional profiles in *Drosophila*, the authors listed all the age-related GO terms that were significantly enriched and classified them based on the tissue where they were expressed and on their directional expression. We found that 106 GO terms were upregulated in fire ant haplo queens and old *Drosophila*, while only 36 were shared between old flies and pleo: however, the difference was not statistically significant (Fisher's Exact Test: P = 0.67). When we compared downregulated GO terms, we found that 11 were shared between haplo and old flies while 67 were shared between old flies and pleo: the difference was statistically significant (Fisher's Exact Test: P = 0.0029). Most of these 67 overlapping GO terms (see Table S7 for details) encompassed genes that were regulated in the gut (32) and fat bodies (23), followed by brain (13), muscles (11), malpighian tubules (7) and accessory glands (6).

**Figure 4:**
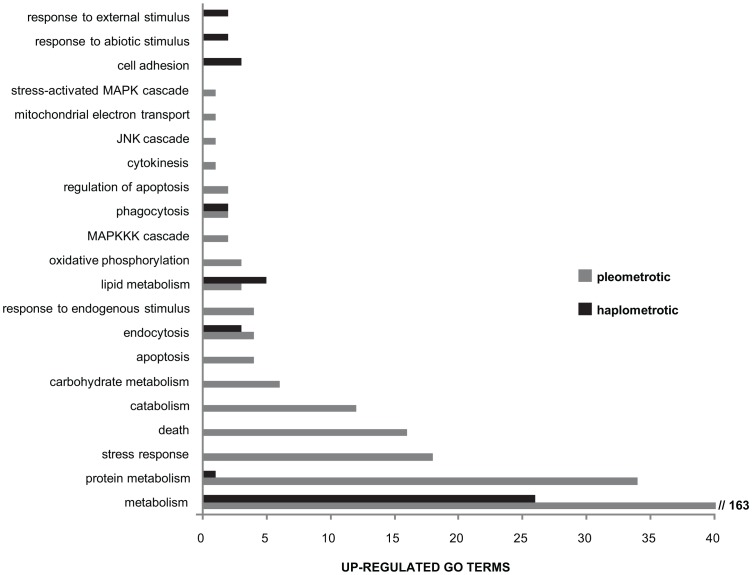
Directional expression of immune-associated GO terms. Overview of larger functional categories encompassing the immune-associated GO terms that were overrepresented among genes upregulated in haplometrotic (grey bars) and pleometrotic (black bars) queens. This result was obtained by overlapping the list of immune system gene classes available in the cateGOrizer with the two lists of significantly enriched GO terms in experiment 1 (Functional Annotation Chart, P<0.05), i.e. cluster 1 for transcripts upregulated in haplometrotic queens and cluster 2 for transcripts upregulated in pleometrotic queens (see Tables S8 and S9, respectively).

Immune-related GO terms were significantly enriched in cluster 1 (genes upregulated in haplo vs. pleo, see Table S4). To better examine the overall expression profiles of genes involved in immune pathways, we obtained a list of significantly enriched GO terms for both cluster 1 and cluster 2 (Functional Annotation Chart in DAVID, P<0.05, see Tables S8 and S9) and we mapped these lists to the list of “Immune system gene classes” available on the CateGOrizer website (http://www.animalgenome.org/bioinfo/tools/catego/slims.html). Thereafter, we compared the relative proportions of the parent/ancestor immune categories between the two groups (Figure 4). This analysis confirmed a significant overrepresentation of immune-related classes in cluster 1 relative to cluster 2 (Nominal Logistic Fit: df = 1, ChiSquare = 61.16, P<0.0001), clearly visible in terms of total number of immune categories present and number of GO terms within common categories. These results suggest that haplometrotic queens overall have higher expression levels of immune-related genes and therefore may be better equipped in terms of immune response.

Next, we examined the expression of the fire ant orthologs of the 177 canonical immune-related genes annotated in honey bees [48]. Orthologs for 83 of these genes were included in our array; 82 were expressed at high enough levels to be included in the analysis, and 34 were within our list of 3003 significantly differentially regulated transcripts (Figure 3B). Expression levels of these genes are not strongly coordinated, with similar numbers of up- vs. downregulated genes in haplo vs. pleo queens. Several genes in the Immune-deficiency (IMD) pathway were differentially regulated, including *Inhibitor of apoptosis 2* (*Iap2*), *TGF-beta activated kinase 1* (*Tak1*), *immune deficiency* (*imd*), *bendless* (*ben*) and *Death related ced-3/Nedd2-like protein* (*Dredd*). Furthermore, several members of the Immunoglobulin (IG) Superfamily were differentially regulated, including *bent* (*bt*), *turtle* (*tutl*), *sidekick* (*sdk*) and *Down syndrome cell adhesion molecule* (*Dscam*).

### Experiment 1: Validation of candidate gene expression levels using quantitative real-time PCR

We examined gene expression levels of candidate genes that were included in one or more GO terms that were significantly enriched in our GO analyses (Figure S3 and Table S10). Expression patterns of all 13 candidate genes were consistent with what observed for haplo and los in the arrays, and these expression differences were significant for 11 genes. We validated *Indy* and *Sod2* for determination of adult life span. In the arrays, *Indy* was downregulated in haplo and *Sod2* was upregulated in haplo: qRT-PCR analysis confirmed these trends and the difference between groups was statistically significant for both genes (P<0.001). For immune response, we validated *Dredd* and *kay*, which were both upregulated in haplo in the arrays: qRT-PCR analysis confirmed this trend and the difference between the two groups of queens was statistically significant for *kay* (P<0.05) but not for *Dredd* (P = 0.29). *Desat1*, *ifc* and *putative fatty acyl-CoA reductase CG5065* were analyzed for their involvement in the synthesis and metabolism of fatty acids. In the arrays, *desat1* and *putative fatty acyl-CoA reductase CG5065* were downregulated in haplo while *ifc* was upregulated in haplo: these trends were confirmed by qRT-PCR analysis and the difference in the expression levels was statistically significant for *putative fatty acyl-CoA reductase CG5065* (P<0.001) and *ifc* (P<0.05) but not for *desat1* (P = 0.11). Reproductive genes included *br* and *Btk29A*, both upregulated in haplo in the arrays: this was confirmed by qRT-PCR (P<0.001). We validated *Sema-5c* and *Mer* because they play a role in olfactory behavior: these genes were downregulated in haplo in the arrays and in the qRT-PCR analysis (P<0.001 and P<0.05, respectively). Finally, we analyzed *fru* for aggressive behavior and *woc* for neurogenesis: these genes were upregulated in haplo in the arrays and in the qRT-PCR analysis (P<0.05 and P<0.001, respectively).

### Experiment 2: Effect of the manipulation of social rank on gene expression patterns

**Figure 5:**
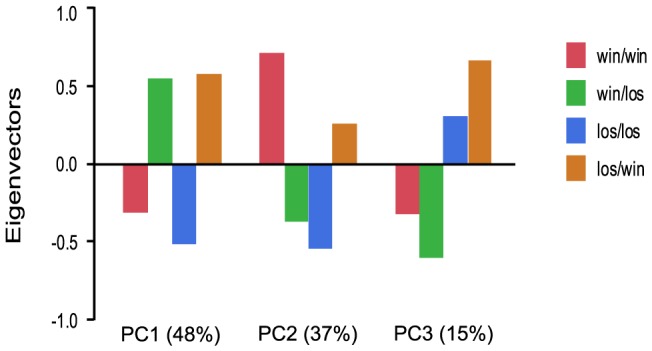
Analysis of global gene expression in pleometrotic queens after manipulation of social rank. Principal Component Analysis of 606 significantly differentially regulated transcripts revealed three principal effects corresponding to switch of the social rank (48%), final rank (37%) and initial rank (15%). win/los = winners switched to losers; win/win = continuing winners; los/win = losers switched to winners; los/los = continuing losers.

We further examined the role of social rank on gene expression patterns by manipulating social rank of individuals in pleometrotic pairs. We swapped winners and losers between groups to generate four groups of queens: winners switched to losers (win/los), losers switched to winners (los/win), continuing winners (win/win) and continuing losers (los/los). Very few transcripts were differentially regulated among these groups, with a total of 616 transcripts at a relatively low significant threshold (FDR<0.1, see Table S11 for a listing of these transcripts). Principal components analysis demonstrated that 48% of the variation in gene expression was associated with switching social rank (win/los and los/win were clustered relative to win/win and los/los), 37% of the variation was associated with the final rank (i.e., win/los and los/los were clustered), while 15% was associated with the initial rank (i.e. win/win and win/los were clustered; Figure 5).

We performed GO analysis with Functional Annotation Clustering on the 527 differentially regulated transcripts that have *Drosophila* orthologs with FlyBase annotations. 21 GO terms were significantly enriched at p-value<0.05 and three survived Benjamini correction: cellular metabolic process, cellular ketone metabolic process and maintenance of protein location (see Table S12). Among the other GO terms, of particular interest was lipid metabolic process, which includes several genes involved in the metabolism of fatty acids such as *Helix loop helix protein 106* (*HLH106*) [49], *scully* (*scu*) [50] and two *putative fatty acyl-CoA reductases*. Additional genes with significant differences in expression included *Coenzyme Q biosynthesis protein 2* (*Coq2*), which plays a role in the response to pathogens, aging and in the insulin-signaling pathway [51], [52], *juvenile hormone acid methyltransferase* (*jhamt*), which was also significantly differentially regulated between win and los in experiment 1 (see above), and *radish* (*rad*), which is involved in learning and memory [53]. Finally, the GO term “response to stress” was significantly overrepresented, which includes key immune response genes such as *immune response deficient 5* (*ird5*) [54], *Ras-related protein Rac1* (*Rac1*) [55], *Hemolectin* (*Hml*) [56], *Argonaute 2* (*AGO2*) [57] and *caspar* (*casp*) [58].

Ninety-three transcripts were differentially regulated both between win and los in experiments 1 (548 transcripts, FDR<0.1) and in experiment 2 (616 transcripts, FDR<0.1): this is significantly more than expected by chance (Hypergeometric Test: Representation factor: 2.2, p<1.009^−13^). These transcripts corresponded to 80 *Drosophila* orthologs, which were used to perform a GO analysis using Functional Annotation Clustering: 6 GO terms appeared to be significantly enriched, including lipid metabolic process (P<0.001) and regulation of hormone levels (P<0.05; Table S13). The expression patterns of the 9 differentially regulated transcripts involved in lipid metabolic process across the two experiments are shown in Table 2.

**Table 2:** Patterns of expression of transcripts associated with lipid metabolic process that were significantly differentially regulated in both experiment 1 and experiment 2 at FDR<0.1.

Sinv_gene	Dmel_ortholog	haplo	win	los	win/win	win/los	los/los	los/win
SI2.2.0_00651	*Inositol-3-phosphate synthase*	up	↓	↓	↓	up	up	↓
SINVlc3_024465	*Putative inositol monophosphatase 3*	up	↓	↓	↓	up	↓	up
SINVlc3_042741	*Putative fatty acyl-CoA reductase CG8306*	up	↓	↓	up	up	↓	up
SI2.2.0_03193	*Dmel_CG31522*	up	↓	up	↓	up	up	↓
SI2.2.0_05591	*Glycosyltransferase 25 family member*	up	↓	↓	↓	up	↓	up
SI2.2.0_03336	*Tafazzin homolog*	↓	up	up	up	↓	↓	↓
SI2.2.0_06292	*juvenile hormone acid methyltransferase*	↓	↓	up	↓	up	up	↓
SI2.2.0_04120	*Putative fatty acyl-CoA reductase CG5065*	↓	up	up	↓	up	up	↓
SI2.2.0_00216	*Dmel_CG17374*	↓	up	up	↓	up	up	up

haplo = haplometrotic queens; los = pleometrotic losers; win = pleometrotic winners; win/los = winners switched to losers; win/win = continuing winners; los/win = losers switched to winners; los/los = continuing losers.

## Discussion

This study demonstrates that social environment greatly influences gene expression in founding queens of the fire ant *Solenopsis invicta*, and that the effects of social rank are secondary. Social environment in the first experiment (haplometrosis vs. pleometrosis) strongly influenced expression of thousands of genes, and the difference between pleometrotic winners and losers was much smaller. However, in terms of gene expression differences, pleometrotic winners were more similar to haplometrotic queens, suggesting that reproductive and social rank still does impact, albeit more subtly, gene expression patterns. In the second experiment, we manipulated both the social environment and social rank of queens in pleometrotic pairs. Switching social rank significantly affected gene expression patterns more than the initial or final social rank of the individual. Several categories of genes were differentially regulated among these groups of queens, including genes involved in core processes such as metabolism, aging and immunity.

In a recent study Ferreira et al. [59] explored the genetic basis of the early phases of social evolution in a primitively eusocial *Polistes* wasp. These authors found that 75% of the 2,442 genes differentially expressed between queen and worker phenotypes were novel, having no significant similarity with described sequences. This result supports the hypothesis that novel genes are likely important for eusocial evolution, as previously suggested by other studies [60], [61]. Interestingly, within our pool of 9,388 genes initially analyzed for experiment 1 (haplometrotic vs. pleometrotic queens), 41% were novel but this percentage decreased to 26% (which is a significantly smaller percentage, Fisher's Exact Test, P<0.0001) when we consider only the genes that were differentially regulated between the two phenotypes of founding queens. Thus, while caste differences in *Polistes* may be associated with novel genes, plasticity in founding behavior in fire ants seems to rely predominantly on more conserved genes.

### Haplometrotic vs. pleometrotic queens

Differences in gene expression between haplometrotic and pleometrotic queens were likely due to differences in the physiological demands placed on singly- vs. multiply-founding queens and differences in the costs associated with social environment, where pleometrotic queens are more likely to incur in higher levels of stress due to the establishment of social ranks. We found that genes involved in core physiological processes, including metabolism, cellular processes, development, morphogenesis and biosynthesis were significantly differentially regulated between these groups of queens. Haplometrotic queens produce more eggs and lose more weight than pleometrotic queens during the claustral period of colony founding [20]: this seems to be due to queen-queen reciprocal reproductive inhibition and oophagy/cannibalism of larvae in pleometrotic associations [62]. Genes associated with reproductive functions (including development of reproductive tissues and production of oocytes and eggs) were upregulated in haplometrotic queens. Furthermore, in order to produce eggs, newly mated queens degrade wing muscle tissues and metabolize fat body storage proteins to produce free amino acids [63]. We found 58 protein-related GO terms and 10 amino acid-related that were upregulated in haplometrotic queens versus 5 and 0, respectively that were upregulated in pleometrotic queens (Functional Annotation Chart, see Tables S8 and S9).

Genes associated with stress response were differentially regulated between haplo and pleo queens. Stress tolerance may be achieved by reducing the production of reactive oxidant species (via improved regulation of mitochondrial processes) and/or by increasing the antioxidant activity [64], [65]. In our study, we found that two mitochondria-related GO terms, namely mitochondrial electron transport, NADH to ubiquinone (15 genes) and mitochondrion organization (18 genes) were upregulated in haplo and none in pleo. Moreover, 9 antioxidant genes were upregulated in haplo, including two superoxide dismutases (*Sod2* and *CCS*), two *Peroxiredoxins* (*6005* and *5037*), *Glutathione S transferase S1* and *PTEN-induced putative kinase 1* (*Pink1*), which plays an essential role in maintaining neuronal survival by preventing neurons from undergoing oxidative stress [66]. These results suggest that haplo queens may experience lower levels of oxidative stress either by producing less ROS or by keeping the levels of antioxidants high. Higher stress levels in pleo queens could be correlated to their social environment, dominated by queen-queen aggressive interactions and competition. Stress tolerance is positively correlated with lifespan [67] and this trait has been used as a proxy for long-lived phenotypes in studies that examine the genetic basis of lifespan [68]. Only *SOD* was upregulated in pleo. Interestingly, overexpression of *SOD* has been correlated to increased organismal longevity in *Drosophila* [69], but this was not confirmed in *Lasius niger*, where long-lived queens expressed lower levels of this gene than short-lived males and workers [70]. It is evident that the effect of *SOD* on longevity is highly dependent upon the sex and genetic background [71] and also the social environment [72].

The overrepresentation of GO terms associated to biosynthesis and metabolism (in particular those related to lipids) prompted us to look closer at the nutritional state of founding queens. Nutrition is closely linked to fertility and longevity [73]. In insects, the insulin-signaling pathway regulates nutrient-sensing [74] while juvenile hormone and ecdysone mediate reproductive processes [75]. In honey bees, long-lived queens have low levels of insulin and juvenile hormone, while they have high levels of FOXO, vitellogenin and ecdysone; opposite patterns are found in sterile short-lived workers [76]. Our results show that haplo had higher levels of FOXO and of the ecdysone receptor. Haplo queens also presumably had lower levels of JH, since levels of *juvenile hormone acid methyltransferase* (*jhamt*), an enzyme that converts inactive precursors of JHs to active JHs [41], were downregulated, and levels of *juvenile hormone epoxide hydrolase 2* (*Jheh2*), involved in juvenile hormone catabolic process, were upregulated.

There is no clear prediction about which group of queens should have a longer life-span. Our analyses show that a large set of aging-related GO terms was upregulated in haplometrotic queens, while a smaller set was upregulated in pleometrotic queens. This result is not sufficient to establish which group of queens is expected to have longer life-span, since ageing is a quantitative trait determined by both environmental and genetic components. Previous studies of the genetics of longevity in *Drosophila melanogaster*, identified sets of genes in which upregulated expression either accelerates or decelerates the aging process [65]. However, in our study, genes from both categories were equally up- and downregulated across haplo and pleo queens (see Figure 3A). Therefore, the knowledge of the genetics of longevity in the insect model *D. melanogaster* cannot be transferred directly to our study system.

Immune-related genes were overexpressed in haplometrotic vs. pleometrotic queens. Most of the overrepresented immune-related GO terms were associated to cellular immunity: endocytosis, phagocytosis, cell adhesion, apoptosis, cytokinesis, the cascade regulating mitogen-activated protein kinase (MAPKKK) and the c-Jun amino-terminal protein kinase (JNK) cascade. In particular, the JNK pathway controls the rapid up-regulation of cytoskeletal genes in response to infection and plays a major role in wound healing [77]. Key genes in the JNK pathways [78] were upregulated in haplo, namely *kayak*, *hemipterous*, *misshapen*, *anterior open* and *Cdc42*. Hemopoiesis is the process that is responsible for production and differentiation of immune cells [79]: two key genes involved in this process, *serrate* and *serpent*, were upregulated in haplo. Haplo queens may have better constitutive immune responses perhaps because they experience less social stress than pleo queens do: in fact, once initial cooperation transitions into open competition, pleo queens frequently engage in reciprocal aggressions which can lead to serious injuries or death. It is hypothesized that there is a trade-off between reproduction, nutrition and immunity [80], suggesting that highly reproductive haplo queens should have overall reduced immune responses during colony foundation period when food sources are limited. However, previous studies in honey bees demonstrated that reproductive queens have higher expression of immune genes than non-reproductive workers [81], [82], and thus this trade-off may not exist in social insect queens, perhaps because queens have more energy resources than workers.

### Pleometrotic queens: Winner vs. loser

Only 43 transcripts were significantly differentially regulated between winners and losers in couples of pleometrotic queens from experiment 1. Although surprising, this result might be explained by the small phenotypic differences between the two types of queens. Previous studies showed that some phenotypic traits such as head width are weakly correlated with the reproductive investment and survival (hence the rank) of pleometrotic cofoundresses [20]. It has been suggested that the relatively weak association between these parameters stems from selection to maintain cooperation [20]. If phenotypic differences strongly correlate with the chances of surviving, smaller queens with lower fighting abilities would be selected not to cooperate and feed the brood in the colony. Thus, the small differences at the genomic level between winners and losers may reflect selection for a system where differences between cofoundresses is sufficiently small so that all of them have a chance of surviving, and thus an interest to cooperate with unrelated individuals.

The two GO terms that were differentially regulated between winners and losers were related to metabolism of lipids and metabolism of hormones. Four transcripts, *bubblegum*, *desat1*, *Dmel_CG17374* and *Dmel_CG31522*, which function in fatty acid metabolism, were differentially regulated. Long-chain fatty acids are the precursors of cuticular hydrocarbons in insects, which can function as nestmate recognition cues and social pheromones in many insect species (reviewed [83]). Interestingly, *bgm*, which encodes a very long-chain fatty acid CoA ligase [36], was downregulated in losers relative to winners and haplo: thus, this gene may be involved in regulating chemical cues related to dominance. Similarly, *desat1*, which is expressed at higher levels in losers than in winners or haplo functions in pheromonal communication [40]. Altered *bgm* expression has also been associated with infection (and correlated with changes in cuticular hydrocarbon profiles) in honey bees [84], while *desat1* appears to play a role in autophagic responses [85]. Thus, these genes may also be involved in signaling infection, nutrient deprivation or other stress responses.

Behavioral manipulation of the social rank in pairs of pleometrotic queens demonstrated that manipulation of social environment (i.e., conditions in which the social rank of the individual changes) had a much larger effect on gene expression than the initial or final social rank of the individual. Note, however, that all individuals in the study switched social partners, which may have elicited additional (undetected) changes in gene expression. Studies in vertebrates have demonstrated that social interactions and changes in the social environment can be one of the most potent stressors [86]. Indeed, genes associate with ‘response to stress’ were significantly enriched, with a set of 30 transcripts differentially regulated among the four groups of manipulated queens (see Results and Table S12). The effects of restructuring social ranks have not been considered broadly in other species [87], but decreased social rank in dark-eyed junco birds is associated with increased metabolic rates, while increased social rank results in a much lower physiological change [88]. Similarly, for dominant, but not for subordinate, birds there is a measurable metabolic cost to joining a new social group [88].

In both experiments, genes involved in lipid biosynthesis and metabolism were differentially regulated, suggesting that these processes play a key role in mediating fire ant founding behavior and foundress associations. Lipids such as cuticular hydrocarbons play a role in advertising the fertility state in many ant species: these compounds are usually more abundant in reproductive queens and egg-laying workers (reviewed in [89]). Indeed, ‘lipid biosynthetic/metabolic process’ was differentially regulated in haplo vs. pleo and in win vs. los in experiment 1 (Tables S4, S5 and S6) and in experiment 2 (Table S12). These results support the hypothesis that lipids (and in particular fatty acids) are of great importance in regulating social interactions between queens and among nestmates in general. In fire ant pleometrotic associations, the pheromones and nestmate recognition chemicals derived from these fatty acids are most likely an important component of the individual's chemical profile, which is used by nestmate queens to decipher the physiological condition and thus the social rank of the partner.

### Conclusions

We used newly developed genomic tools to examine the gene expression patterns associated with complex social behaviors involved in colony founding by fire ant queens. Our results suggest that social environment (haplometrotic vs. pleometrotic, switched vs. maintained social rank) is more important than the social rank or internal condition of the individual in regulating gene expression patterns, and presumably downstream physiological and behavioral traits. Furthermore, because the process of pleometrotic colony founding in fire ants has all the features of a primitively social system in which morphologically, physiologically, and genetically similar individuals perform cooperative behavior to form social groups of unrelated individuals, this is an excellent model to examine the genes that underlie these social behaviors. We found that several core processes were significantly differentially regulated, including metabolism, stress response, aging, reproductive processes, and immunity. Interestingly, lipid metabolic processes were regulated across experiments; these may play a role in both nutrient storage/mobilization and chemical communication. In the future, it will be interesting to investigate whether the molecular pathways characterized in this study also are operating at earlier stages of the co-founding process (e.g., before the emergence of workers). Such studies will help elucidate the mechanisms responsible for the transition from cooperation to conflict in pleometrotic founding queens. Finally, fire ants also display genetically distinct monogyne (colonies headed by a single queen) and polygyne (colonies headed by multiple queens) social forms. It will be of great interest to determine if the same genes that regulate haplometrosis and pleometrosis also are involved in regulating queen number in mature colonies.

## Materials and Methods

### Insect collection and rearing

A total of 787 fire ant queens were collected immediately after a nuptial mating flight on May 5^th^, 2010 in a large parking lot (Target, 3970 SW Archer Rd, Gainesville, FL) and weighed. Since the area of collection has a high prevalence of monogyne colonies, we expected these queens to belong to the monogyne social form; this was subsequently confirmed by screening 108 queens for social form using *Gp-9* as a marker following the protocol as described in [90]. The remaining 679 queens were split into two groups: 308 queens were set up in pairs (pleometrosis) based on having similar weights (range ±0.2 mg) and paint-marked with different colors, while 371 queens were set up individually (haplometrosis) and paint-marked as well. All the queens were provided with a nesting chamber consisting of a glass tube half-filled with water, which was covered by a cotton ball and a layer of dental plaster: this keeps the chamber moist but avoids an excess of water which is deleterious for the young brood. Tubes were sealed with a loose cap to provide air flow. Specimens were reared in the dark at 28°C, 70% relative humidity under claustral conditions (no food and no water) for 1 month.

### Experiment 1: Effect of social environment and social rank on gene expression patterns

After eclosion of the first batch of workers (minims), incipient colonies were provided with water, sugar water and frozen crickets. Glass tubes were set open in pencil boxes coated with Fluon to prevent escape. Queens were subsequently monitored daily until it was possible to identify the social rank of the two cofoundresses in pleometrotic couples. Previous studies have found that the initial cooperation between the two cofoundresses turns into conflict after the emergence of minims, resulting in the execution of one queen [19]. Queens that survive the competition (winners) are usually located at the top of the brood pile within the nest chamber and they are generally tended by workers; conversely, queens that will be executed (losers) are normally seen outside the nest chamber, hiding from workers in order to avoid being attacked (Figure S4). We used these observations to establish the social rank of the two pleometrotic queens, i.e. winner and loser. We collected 25 pleometrotic couples and 25 haplometrotic queens in dry ice and stored them at −80°C to be later processed.

### Experiment 2: Effect of the manipulation of social rank on gene expression patterns

This assay was performed with 34 couples of pleometrotic queens from the same pool of newly mated queens as experiment 1. The queens were paired and placed in nesting chambers as before. After emergence of minims, queens' behavior was monitored as before. Once the behavioral observation revealed the social rank of the two cofoundresses, queens were weighed again and re-paired with a different partner. We created the following three groups of queens: a) winner+winner (similar weight), b) loser+loser (similar weight), and c) winner+loser (different weights). Again, we monitored the behavior until the social rank of the newly coupled specimens was evident and we collected 4 new behavioral phenotypes in the same way as above: a) winners switched into losers (win/los, N = 7), b) losers switched into winners (los/win, N = 11), c) continuing winners (win/win, N = 12) and d) continuing losers (los/los, N = 5).

### Sample preparation for molecular analyses

Individual fire ant queens were thawed and dissected under cold RNAlater (Qiagen, Valencia, CA) to confirm the mating status: unmated queens were not included in the analysis. Total RNA was extracted using the RNeasy Plus kit (Qiagen) combined with a RNase-Free DNase step (Qiagen) to remove any possible contamination by genomic DNA. Subsequent steps in the microarray analysis were performed at the Penn State Genomic Core Facility. RNA concentration and purity were assessed using NanoDrop and Qubit and RNA quality was assessed using RNA Nano Chips on the Agilent Bioanalyzer. 1 µg of each sample was amplified using the Ambion (Life Technologies) Amino Allyl MessageAmp II aRNA Amplification Kit (AM1753). 15 µg of aRNA were dyed with Cy3 or Cy5 (GE Health Care #RPN5661) and subsequently purified according to the Ambion Kit instructions. 1.5 µg of a Cy3 labeled sample were combined with 1.5 µg of a Cy5 labeled sample and fragmented using RNA Fragmentation Reagents (Ambion AM8740) according to the manufacturer's instructions. Samples were hybridized with mixing in a MAUI hybridization instrument overnight at 42°C. Arrays were scanned using Axon GenePix 4000B.

For the first microarray developed to validate the efficiency of probe sequences, we pooled RNA samples (2 µg total) from different castes, developmental instars and social forms as follows: 3 female alates, 15 workers, 5 larvae and 5 pupae from both monogyne and polygyne social forms and 5 males from monogyne colonies only.

### Microarray design and validation

The fire ant genome includes an official gene set of 16,569 protein-coding genes that were generated by a combination of *ab initio*, EST-based, and sequence similarity-based methods [27]. For our microarray studies, we combined the official gene set with a set of ESTs obtained from assemblies of the fire ant transcriptome for a total set of 63,436 sequences (“transcripts”). We successfully designed 60-mer probes for 51,531 of these transcripts (Roche NimbleGen, Inc., Madison WI). These sequences/probes were grouped into four categories: ESTs with gene models (EWGM, 7433 transcripts), ESTs without gene models (EWOGM, 40,613), gene models (GM, 3246) and gene models redundant with other models (GMRWOM, 239).

We developed and used a first microarray (1-plex 385,000 probe capacity, Roche NimbleGen, Inc., Madison WI) to validate the probe design and test multiple probes per transcript. On average, we designed 7 probes per transcript for a total of 355,930 probes. Each probe was tested for both the red (Cy5) and the green (Cy3) dyes. For transcripts with only one probe (N = 296), we verified that the probe had acceptable intensities for both dyes. For the other transcripts we examined the performance of the probes with the green dye only, because these showed consistently higher intensity compared to the red dye. Probes were ranked in the follow manner: a) if there were only 2 probes per transcript (N = 230), we selected the one with higher intensity; b) if there were 3 to 6 probes (N = 744), we calculated the ratio “probe intensity/median intensity of all probes for that transcript” and selected the probe with highest ratio if the value was <3, otherwise we selected the probe with the second highest ratio; c) for transcripts with 7 probes (N = 50,261), we followed the procedure as in “b” but, in case the probe with the highest ratio was >3, we removed that probe, calculated new ratios and selected a new probe with highest ratio. This procedure allowed us to select the probes with highest intensity that were not outliers.

Selected probes were printed in pairs on two 12-plex microarrays (each array had a 135,000 probe capacity, Roche NimbleGen, Inc., Madison WI). We used a loop design with dye swaps incorporated, allowing us to hybridize 24 RNA samples to each array. For experiment 1 we hybridized 8 haplometrotic queens, 8 pleometrotic winners and 8 pleometrotic losers (Figure S5) and for experiment 2 we compared 6 win/los, 6 los/win, 6 win/win and 5 los/los (Figure S6).

### Analysis of gene expression

Any spots with an intensity of less than 300 (the background level on the arrays) were removed from the analyses, as were spots present on less than 20 out of 24 arrays. Expression data were log-transformed and normalized using mixed-model normalization (proc MIXED, SAS, Cary, NC) with the following model:
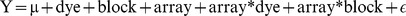
where Y is expression, dye and block are fixed effects, and array, array*dye and array*block are random effects. Transcripts with significant expression differences between groups were detected by using a mixed-model ANOVA with the model:
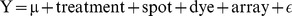
where Y represents the residual from the previous model. Treatment, spot and dye are fixed effects and array is a random effect. P-values were corrected for multiple testing using a false discovery rate of <0.001 for experiment 1 and <0.1 for experiment 2 (proc MULTTEST, SAS). Because the number of differentially regulated transcripts for experiment 1 was very high (∼13,000 out of ∼50,000), and to avoid an excess of redundancy among the different groups of transcripts, we included only probes corresponding to GM and EWGM (see above).

Hierarchical clustering, using the Ward method, and principal component analysis (PCA) for global patterns of gene expression were performed in JMP 9.0.2 (SAS, Cary, NC). We used Genesis 1.7.6 (Graz, Austria) to cluster differentially regulated genes based on average linkage and to perform k-means clustering in experiment 1. Gene Ontology analysis was performed using functional annotation chart/clustering in DAVID version 6 [28], [29] using DAVID default population background and a cutoff of p<0.05. For all Gene Ontology (GO) analyses, fire ant genes were matched to their *Drosophila* orthologs in FlyBase (http://flybase.org/). CateGOrizer [31] was used to count the occurrences of significantly enriched GO terms within each of the pre-defined set of parent/ancestor GO terms. The array data were deposited on the ArrayExpress website according to MIAME standards (ArrayExpress accession: E-MEXP-3886 for experiment 1, E-MEXP-3898 for experiment 2).

### Comparative studies

We compared the results from experiment 1 to the following studies:

1. experiment 2 from this study: we overlapped 548 transcripts that were significantly differentially regulated at FDR<0.1 between win and los in experiment 1 to all the significantly differentially regulated transcripts from experiment 2 (606, FDR<0.1).
2. aging in *Drosophila*: we compared directional expressions for all the significantly enriched GO terms obtained from Functional Annotation Chart for experiment 1 and the significantly enriched GO terms that were associated to aging in the fruit fly based on Zhan et al. [47].

We performed overlaps between list of transcripts and GO terms with Venny [91]. In the first comparative study we overlapped fire ant transcripts directly, while in the second study we used *Drosophila* orthologues (FlyBase numbers) to compare fire ant transcripts to the genes of the fruit fly. Statistical significance of the overlap was calculated using a hypergeometric test (http://nemates.org/MA/progs/overlap_stats.html). Selected GO analyses based on study overlap were performed in DAVID as above. In the second study, to test for the significant agreement in the patterns of expression between two studies we performed Fisher's Exact Tests in JMP.

### Validation of candidate gene expression using Quantitative Real-Time PCR

We examined gene expression levels of the following candidate genes (Table S10): *Indy* and *Sod2* (determination of adult life span); *Dredd* and *kay* (immune response); *desat1*, *ifc* and *Putative fatty acyl-CoA reductase CG5065* (synthesis and metabolism of fatty acids); *br* and *Btk29A* (reproductive functions); *Sema-5c* and *Mer* (olfactory behavior); *fru* (aggressive behavior) and *woc* (neurogenesis). We used the total RNA extracted from fire ant queens for the microarray analysis and compared gene expression between haplo and los on an ABI Prism 7900 sequence detector (Applied Biosystems, Foster City, CA, USA). cDNA was made using SuperScript III First-Strand Synthesis System for RT-PCR (Invitrogen-Life Technologies, Carlsbad, CA, USA) and Random Hexamers according to the manufacturer's protocol. The cDNA was then diluted 2(x) with ultra-pure water. Amplification was performed in a 10 µl reaction mixture containing 5 µl of 2× SYBR Green Master Mix (Applied Biosystems-Life Technologies, Carlsbad, CA, USA), 1 µl of each primer (10 µM) and 2 µl of cDNA at the following conditions: 50°C for 2 min, 95°C for 10 min, 40 cycles of 95°C for 15 sec and 60°C for 1 min, a dissociation step of 95°C for 15 sec and 60°C for 15 sec. We used 8 queens per group: triplicate reactions were performed for each of the samples and averaged for use in statistical analysis. Expression levels of candidate genes were normalized to the geometric mean of two housekeeping genes, *Rp-9* and *Rp-37* [27]. Negative control (cDNA reaction without RT enzyme) was also used. Primer sequences were developed in Primer3Plus (http://www.bioinformatics.nl/cgi-bin/primer3plus/primer3plus.cgi) and primer efficiency was first validated using standard curves. Statistical analysis was performed with nonparametric Kruskall-Wallis rank sums in JMP 10 (SAS, Cary, NC). The data were shown normalized to the haplo group.

## Supporting Information

Figure S1 Transcripts upregulated in haplometrotic queens. Expression patterns of 2280 significantly differentially regulated transcripts grouped in cluster 1 by k-means clustering in Genesis (for a GO analysis of these transcripts see Table S4).
(EPS)

Figure S2 Transcripts downregulated in haplometrotic queens. Expression patterns of 912 significantly differentially regulated transcripts grouped in cluster 2 by k-means clustering in Genesis (for a GO analysis of these transcripts see Table S5).
(EPS)

Figure S3 Quantitative real-time PCR validation of expression levels of genes of interest. Expression levels of the following genes associated with GO terms of interest were analyzed using quantitative real-time PCR (see Table S10 for detailed information about these genes and the primers we used): *Indy* and *Sod2* (determination of adult life span); *Dredd* and *kay* (immune response); *desat1*, *ifc* and *Putative fatty acyl-CoA reductase CG5065* (synthesis and metabolism of fatty acids); *br* and *Btk29A* (reproductive functions); *Sema-5c* and *Mer* (olfactory behavior); *fru* (aggressive behavior) and *woc* (neurogenesis). Mean expression levels in losers were normalized to levels of expression in haplometrotic queens. Each sample group consisted of 8 individuals; these 8 individuals were also used in the microarray analysis. Statistical analysis was performed with Kruskall-Wallis rank sums: * = P<0.05; ** = P<0.01; *** = P<0.001. #For a better visualization of the results, the bar associated to the gene *Putative fatty acyl-CoA reductase CG5065* is not represented in full length in losers: average relative expression for this gene was 14.06 with S.E. ±0.35.
(EPS)

Figure S4 Behavioral observation of pleometrotic couples. Just before the emergence of the first workers, haplometrotic queens and pleometrotic couples were placed in pencil boxes where it was easier to observe queen-queen and queen-workers interactions. As shown in the figure, in pleometrotic couples the winner queen was usually found inside the nest chamber (glass tube, where the eggs and the brood are) while the loser was find outside the nest chamber, frequently hiding in order to avoid any contact with the winner or the workers.
(TIF)

Figure S5 Microarray hybridization scheme for experiment 1. For each group of queens, 8 individuals where hybridized in a loop design: 4 individuals were labeled with the Cy3 dye and other 4 with the Cy5 dye. 12-plex array slides with 135,000 probe capacity where designed by Roche NimbleGen, Inc. (Madison WI). haplo = haplometrotic queens; los = pleometrotic losers; win = pleometrotic winners.
(EPS)

Figure S6 Microarray hybridization scheme for experiment 2. For each group of queens, 6 individuals where hybridized in a loop design: 3 individuals were labeled with the Cy3 dye and other 3 with the Cy5 dye. 12-plex array slides with 135,000 probe capacity where designed by Roche NimbleGen, Inc. (Madison WI). win/los = winners switched to losers; win/win = continuing winners; los/win = losers switched to winners; los/los = continuing losers.
(EPS)

Movie S1 Movie clip showing the occurrence of aggressive interactions among cofoundresses after the emergence of workers. Note the difference between pair #9, where pleometrotic queens clearly perform fighting behavior and the two other pairs of cofounding queens, where the interactions are still cooperative.
(WMV)

Table S1 Experiment 1: significantly differentially regulated transcripts (P<0.001).
(XLSX)

Table S2 Experiment 1: significantly enriched GO terms and KEGG pathways (Functional Annotation Chart, P<0.05).
(XLSX)

Table S3 Experiment 1, output of the cateGOrizer analysis: representation of GO_slim2 ancestor terms.
(XLSX)

Table S4 Experiment 1, k-means clustering, cluster 1 (genes upregulated in haplo): significantly enriched GO terms (Functional Annotation Clustering, medium stringency, P<0.05).
(XLSX)

Table S5 Experiment 1, k-means clustering, cluster 2 (genes downregulated in haplo): significantly enriched GO terms (Functional Annotation Clustering, medium stringency, P<0.05).
(XLSX)

Table S6 Experiment 1: GO terms significantly enriched (Functional Annotation Clustering, medium stringency, P<0.05) and significantly differentially regulated transcripts (P<0.001) between winners and losers.
(XLSX)

Table S7 Comparative studies: GO terms encompassing significantly downregulated genes that were in common between experiment 1 and old *Drosophila* in Zhan et al. [47].
(XLSX)

Table S8 Experiment 1, cluster 1: significantly enriched KEGG pathways and GO terms that were used in the analyses with the cateGOrizer “Immune system gene classes” (Functional Annotation Chart, P<0.05).
(XLSX)

Table S9 Experiment 1, cluster 2: significantly enriched KEGG pathways and GO terms that were used in the analyses with the cateGOrizer “Immune system gene classes” (Functional Annotation Chart, P<0.05).
(XLSX)

Table S10 Quantitative real-time PCR validation of expression levels of genes of interest from experiment 1: gene lists and primers' sequences.
(XLSX)

Table S11 Experiment 2: significantly differentially regulated transcripts (P<0.1).
(XLSX)

Table S12 Experiment 2: significantly enriched GO terms (Functional Annotation Clustering, medium stringency, P<0.05).
(XLSX)

Table S13 Comparative studies: GO analysis of overlapping transcripts that were significantly differentially regulated (P<0.1) between win and los in experiment 1 and experiment 2.
(XLSX)
