# Supplementary figures and images for: Sociogenomics of Cooperation and Conflict during Colony Founding in the Fire Ant Solenopsis invicta
Source: PLoS Genet. 2013 Aug 8;9(8):e1003633. doi: 10.1371/journal.pgen.1003633 (PMC3738511; doi:10.1371/journal.pgen.1003633)

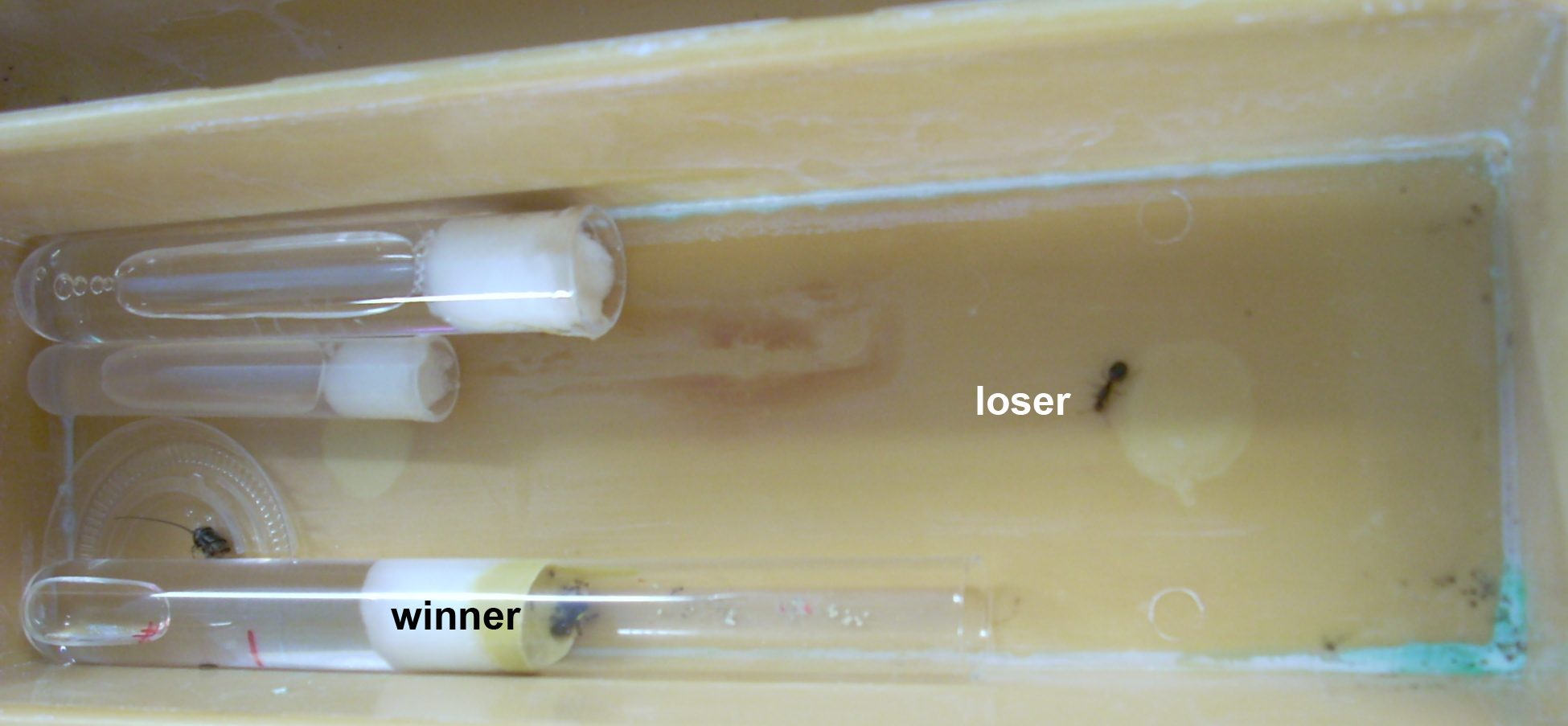

Supplement: Figure S4 — Behavioral observation of pleometrotic couples. Just before the emergence of the first workers, haplometrotic queens and pleometrotic couples were placed in pencil boxes where it was easier to observe queen-queen and queen-workers interactions. As shown in the figure, in pleometrotic couples the winner queen was usually found inside the nest chamber (glass tube, where the eggs and the brood are) while the loser was find outside the nest chamber, frequently hiding in order to avoid any contact with the winner or the workers. (TIF) [file pgen.1003633.s004.tif]
